# Protection from Hemolytic Uremic Syndrome by Eyedrop Vaccination with Modified Enterohemorrhagic *E. coli* Outer Membrane Vesicles

**DOI:** 10.1371/journal.pone.0100229

**Published:** 2014-07-17

**Authors:** Kyoung Sub Choi, Sang-Hyun Kim, Eun-Do Kim, Sang-Ho Lee, Soo Jung Han, Sangchul Yoon, Kyu-Tae Chang, Kyoung Yul Seo

**Affiliations:** 1 The Graduate School of Yonsei University, Seoul, South Korea; 2 Department of Ophthalmology, National Health Insurance Service Ilsan Hospital, Goyang city, South Korea; 3 Viral Infectious Disease Research Center, Korea Research Institute of Bioscience and Biotechnology (KRIBB), Daejeon, South Korea; 4 Department of Ophthalmology, Eye and Ear Hospital, Severance Hospital, Institute of Vision Research, Yonsei University College of Medicine, Seoul, South Korea; 5 Brain Korea 21 Project for Medical Science, Yonsei University, Seoul, South Korea; 6 The National Primate Research Center, Korea Research Institute of Bioscience and Biotechnology (KRIBB), Ochang, Cheongwon, Chungbuk, South Korea; Indian Institute of Science, India

## Abstract

We investigated whether eyedrop vaccination using modified outer membrane vesicles (mOMVs) is effective for protecting against hemolytic uremic syndrome (HUS) caused by enterohemorrhagic *E. coli* (EHEC) O157:H7 infection. Modified OMVs and waaJ-mOMVs were prepared from cultures of MsbB- and Shiga toxin A subunit (STxA)-deficient EHEC O157:H7 bacteria with or without an additional *waaJ* mutation. BALB/c mice were immunized by eyedrop mOMVs, waaJ-mOMVs, and mOMVs plus polymyxin B (PMB). Mice were boosted at 2 weeks, and challenged peritoneally with wild-type OMVs (wtOMVs) at 4 weeks. As parameters for evaluation of the OMV-mediated immune protection, serum and mucosal immunoglobulins, body weight change and blood urea nitrogen (BUN)/Creatinin (Cr) were tested, as well as histopathology of renal tissue. In order to confirm the safety of mOMVs for eyedrop use, body weight and ocular histopathological changes were monitored in mice. Modified OMVs having penta-acylated lipid A moiety did not contain STxA subunit proteins but retained non-toxic Shiga toxin B (STxB) subunit. Removal of the polymeric O-antigen of O157 LPS was confirmed in waaJ-mOMVs. The mice group vaccinated with mOMVs elicited greater humoral and mucosal immune responses than did the waaJ-mOMVs and PBS-treated groups. Eyedrop vaccination of mOMVs plus PMB reduced the level of humoral and mucosal immune responses, suggesting that intact O157 LPS antigen can be a critical component for enhancing the immunogenicity of the mOMVs. After challenge, mice vaccinated with mOMVs were protected from a lethal dose of wtOMVs administered intraperitoneally, conversely mice in the PBS control group were not. Collectively, for the first time, EHEC O157-derived mOMV eyedrop vaccine was experimentally evaluated as an efficient and safe means of vaccine development against EHEC O157:H7 infection-associated HUS.

## Introduction

Enterohemorrhagic *E. coli* (EHEC) can cause severe diarrhea, hemorrhagic colitis, which is often accompanied by hemolytic anemia, thrombocytopenia, and acute renal failure, which are the hallmarks of hemolytic uremic syndrome (HUS) [1]. Although typical EHEC strains are classified by the production of Shiga toxin (STx) and the possession of a locus of enterocyte effacement (LEE) in the chromosome, atypical EHEC lacking LEE pathogenicity islands can be associated with HUS, as recently witnessed in a German outbreak of *E. coli* O104:H4 [2]. Despite the lethal outbreak of HUS due to *E. coli* O104:H4, EHEC O157:H7 remains the most important causative strain involved in the manifestation of HUS worldwide [3]. Accordingly, the development of effective vaccines preventing EHEC O157:H7 infection-associated HUS are of prime research interest.

Outer membrane vesicles (OMVs) are spherical membrane blebs shed by Gram-negative bacteria [4]. They carry not only native antigens expressed in the outer membrane, but also exogenous protein epitopes [5] and retain self-adjuvanticity that can be exerted by the inclusion of toll-like receptor agonists (lipopolysaccharide [LPS], outer membrane lipoproteins, flagellin, etc.) [6]. Several reports have demonstrated that vaccination with OMVs is sufficient to induce an immune response and protect vaccinated organisms from subsequent pathogen challenge [7]–[10]. However, up until now, an effective and safe OMV vaccine for protection from EHEC O157:H7 infection and sequelae HUS has not been reported, presumably because OMVs generated from EHEC O157:H7 are intrinsically toxic due to presence of STx exotoxin and LPS endotoxin, which are two major virulence factors that contribute towards the development of HUS. In order to overcome the toxicity of the EHEC O157-OMVs, we used a detoxified OMV (produced from MsbB- and STxA-deficient mutant) that was characterized previously for use as a vaccine [5], [11]. Moreover, we generated waaJ-mOMVs, which were composed of a truncated version of O157-LPS lacking the O-antigen side chains, to test whether the absence of O-antigen in LPS would affect immunogenicity of mOMVs administered via an ocular-mucosal route.

We found that eyedrop vaccination with mOMVs of EHEC O157:H7 induced humoral and mucosal immune responses and, without the use of commercial adjuvants, was able to protect immunized mice from further challenge with wtOMVs, which are believed to be produced in the gut of EHEC O157-infected hosts and have been suggested as the causative agent for HUS [12]. We also demonstrated that loss of the O-antigen by truncation of O157-LPS to the core region made waaJ-mOMVs significantly less immunogenic, indicating the LPS O-antigen in the mOMVs plays a significant role in inducing a protective immune response against lethal O157-OMV challenge.

## Materials and Methods

### Preparation of OMVs of EHEC O157:H7 strains

MsbB- and STxA-deficient mutants of EHEC O157:H7 (Sakai-DM/*stx1A*/*stx2A*) [11] were used as parental strains for producing mOMVs. The preparation of OMVs has been described previously [12]. Briefly, a mOMV producing strain was inoculated in 500 ml of LB broth and cultured with agitation overnight at 37°C. The bacteria were then pelleted by centrifugation (12,000×g) for 10 min at 4°C. The resulting supernatant was recovered and further passed through a 0.22 µm pore-size filter. The mOMVs were harvested by concentrating the filtrates using a QuixStand ultra-filtration system (GE Healthcare, Buckinghamshire, UK) equipped with a membrane cartridge (100 kDa cutoff). Next, mOMVs present in the concentrated sample were collected and pelleted by ultracentrifugation at 100,000×g for 2 h at 4°C. The crude mOMV pellet was then resuspended in 3 ml PBS for further OMV purification using a sucrose-gradient ultracentrifugation step as described previously [5].

### Inactivation of the *waaJ* Gene for the production of waaJ-mOMVs

An allelic exchange approach was used to facilitate deletion of *waaJ* in the chromosome of the mOMV producing strain carrying pKD46 using a mutant allele (Δ*waaJ*::Cm) constructed in a pUC18 vector. Briefly, the Δ*waaJ*::Cm allele was constructed by cloning a genomic DNA fragment (∼1.2 kb) encoding the *waaJ* ORF. PCR primers used for amplification of the *waaJ*-encoding DNA region were designated Fw-WaaJ (GAATTGAAA*GAATTC*GGCTATACATATC) and Rv-WaaJ (GATTTGG*AAGCTT*GGTACGCTGAGCAA C), and the resulting DNA product was further manipulated by digestion with *Eco*RI and *Hin*dII restriction enzymes (underlined in the primer sequences, respectively) for insertion into the pUC18 vector digested with the same enzymes. The resulting *waaJ*-pUC18 clone was used as a template DNA for subsequent inverse PCR to delete an internal 430-bp DNA fragment from the *waaJ* ORF cloned in pUC18. Inverse PCR was then carried out with WaaJ-SalF (GTATTTATACTATAAA *GTCGAC*AATTTGAAG) and WaaJ-KpnR (GATTGA*GGTA CC*GCCTTCAATGGCTC) primers to delete the 430-bp DNA fragment and to generate restriction enzyme (*Sal*I and *Kpn*I) sites (underlined, respectively) at both ends of the amplicon. Next, the Cm-cassette from pKD3 DNA obtained by PCR with the FKD3-Sal and RKD3-Kpn primer set [12] was digested with *Sal*I and *Kpn*I, and was used to replace the 430-bp deleted region of the *waaJ* gene. The resulting Δ*waaJ*::Cm allele was introduced into the mOMV-producer strain carrying pKD46 by electroporation to create the Δ*waaJ*::Cm mutant of the mOMV-producer strain, which was designated EHEC O157:H7-waaJ. The resulting mutant (EHEC O157:H7-waaJ) was cultivated to produce the waaJ-mOMV, which contains a truncated (rough) LPS due to loss of WaaJ activity and is required for LPS core oligo-saccharide extension [13].

### Western Blot Analysis

Protein concentrations of OMV samples were estimated using the BCA protein assay kit (Pierce, Rockford, IL) and samples were analyzed by SDS-PAGE (15% gel). Gels were then transferred to a nitrocellulose membrane (Invitrogen, Carlsbad, CA) for Western blot analysis. Monoclonal antibodies against STx2B (Biodesign, Saco, ME) and STx2A (clone 11E10; Santa Cruz Biotech, Dallas, TX) were purchased from a commercial source. Peroxidase-conjugated rabbit anti-mouse IgG (Sigma-Aldrich) was used as the secondary antibody for ECL detection of target proteins.

### Negative Staining for Electron Microscopy

To visualize OMVs, samples were applied to a formvar-coated grid by adsorption. The grid was then stained with 2% uranyl acetate, blotted with filter paper, and dried in air. Samples were then examined under a CM20 TEM microscope (Philips, the Netherlands).

### LPS Preparation and SDS-PAGE Analysis

LPS was prepared on a small scale using the SDS-proteinase K treated whole-cell lysate method adapted from Hitchcock and Brown [14]. Briefly, bacteria were grown on LB plates overnight. Cells were then scraped from the plates, resuspended in PBS, lysed in buffer containing 2% SDS, 4% β-mercaptoethanol, and 1 M Tris (pH 6.8), and boiled for 10 min. The resulting lysates were then treated with proteinase K for 60 min at 60°C followed by a hot-phenol extraction. The phenol-phase containing most of the LPS was recovered, and LPS was precipitated by the addition of methanol and chloroform (1∶2 v/v) followed by centrifugation. The LPS pellet was then suspended in distilled water (DW) or SDS sample buffer and stored at −20°C until use. LPS was separated on a 16% Tricine SDS-PAGE gel (Novex, San Diego, CA) and visualized by silver staining.

### Animals

*This study was performed in strict accordance with the recommendations in the Guide for the* Care and Use Committee of Yonsei University Health System. *The committee* has reviewed and approved the animal study protocol (Approval No: 2011–0261). Specific pathogen-free BALB/c mice, aged 6–10 weeks, were purchased from Charles River Laboratories (Orient Bio, Sungnam, Korea) and were maintained under specific pathogen-free conditions in the experimental facility at Yonsei University Health System where they received sterilized food and water ad libitum. *All surgeries were performed after* sacrificed by CO_2_ narcosis *and every effort was made to minimize suffering.*

### Immunization with mOMVs and waaJ-mOMVs

For ocular immunization, five mice were anesthetized and 10 µg mOMVs or waaJ-mOMVs suspended in 10 µl PBS were dropped on the conjunctival sac of each eye by micropipette. Two weeks after vaccination, mice were boosted by the same method. In additional experiments, 10 µg mOMVs plus 10 µg polymyxin B (PMB) suspended in 10 µl PBS were immunized.

### Sample Collection

After intraperitoneal injection of mice with pilocarpine (500 mg/kg body weight; Sigma-Aldrich), saliva was obtained. Tear and vaginal wash samples were obtained by lavage with 10 µl or 50 µl PBS. Serum was obtained by tail venipuncture. Fecal extracts were obtained by adding weighed feces to PBS containing 0.1% sodium azide followed by vortexing. The mixture was then centrifuged and the supernatants were collected.

### Indirect Enzyme-Linked Immunosorbent Assay (ELISA)

ELISA plates (Falcon, Franklin Lakes, NJ) were coated with mOMVs, waaJ-mOMVs, and wtOMVs, respectively, in PBS and incubated overnight at 4°C. Blocking was performed with 1% BSA (Sigma-Aldrich) in PBS, and 2-fold serial sample dilutions were applied to plates. HRP-conjugated goat anti-mouse IgG or IgA (Southern Biotechnology Associates, Birmingham, AL) was added to each well and incubated overnight at 4°C. A tetramethylbenzidine solution (Moss, Pasadena, MD) was used for color development. Plates were measured at 450 nm using an ELISA reader (Molecular Devices, Sunnyvale, CA) after the addition of stopping solution (0.5 N HCl).

### Protection Assay against Wild-Type OMVs

Four weeks after eyedrop immunization with mOMVs, 1.5X LD_50_ of wild-type EHEC O157:H7 OMVs (LD_50_ 0.274 mg/kg) [11] were injected intraperitoneally for the challenge experiment. Body weight changes were monitored daily for 8 days after injection. For serum sampling by puncturing the retro-orbital area, mice were anesthetized by intraperitoneal injection of ketamine (100 mg/kg body weight) and xylazine hydrochloride (10 mg/kg body weight) *and* serum was obtained daily for 8 days. Blood samples were analyzed for BUN and Cr. For histopathological observation of kidney, a survived mouse from each group was sacrificed on day 4 post challenge. All challenged mice were allowed to proceed to death as a direct result of the wtOMV challenge (we could not employ humane endpoints because it was hard to anticipate accurate death point based on only bodyweight changes, BUN and Cr values. Actually, unvaccinated mice suddenly died with gradual bodyweight loss but acute increase of BUN and Cr values) and all survived mice after the protection assay was finished were sacrificed by CO_2_ narcosis.

### Histology

Eye tissues including the conjunctiva and eye balls from PBS control and mOMV-treated mice or whole kidney tissues from wtOMV challenged mice were washed with PBS and fixed in 4% formaldehyde for 24 h at 4°C. The tissues were dehydrated by gradual soaking in alcohol and xylene gradients followed by embedding in paraffin. Paraffin-embedded specimens were cut into 5-mm sections and stained with H&E.

### Safety Evaluation

In order to confirm the safety of vaccinations, some groups of BALB/c mice were administered with 10 µg of mOMVs or wtOMVs resolved in 5 µl of PBS by eyedrop on each eye or 10 µg of wtOMVs resolved in 100 µl of PBS intraperitoneally. Body weight changes were monitored on a daily basis for 5 days. Eye tissues were acquired at 24, 48, 72 h after administration of 10 µg of mOMVs for histologic examination.

### Statistical Analysis

All data are expressed as the mean ± SD. Statistical analyses were performed by *t*–tests (Sigma plot).

### Ethics Statement

All experiments involving animal subjects were conducted in strict accordance and adherence to relevant national and international guidelines regarding animal handling as mandated by the Institutional Animal Care and Use Committee (IACUC) of Yonsei University Health System (Seoul, Korea).

## Results

### Separation of LPS Molecules Extracted from mOMV Producing Strains

**Figure 1:**
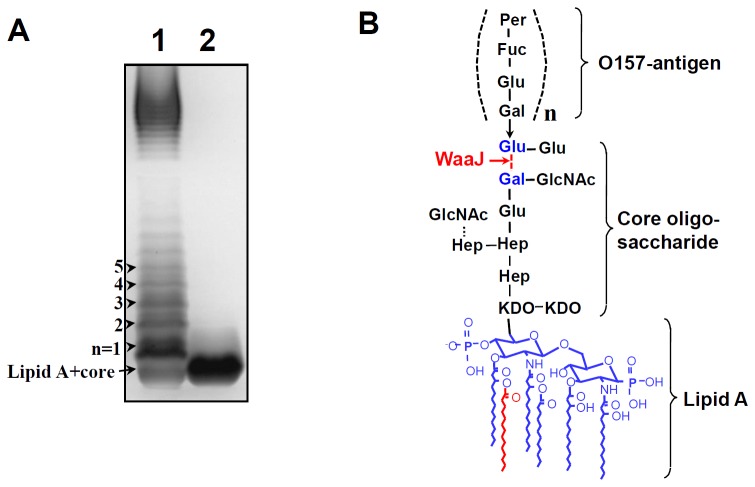
Separation of LPS molecules extracted from mOMV and waaJ-mOMV producing strains. (A) Lanes 1 and 2, silver-stained LPS samples extracted from mOMV producing strains (MsbB- and STxA-deficient mutant of EHEC O157:H7) and waaJ-mOMV producing strains (EHEC O157:H7-waaJ mutant), respectively. Because of the introduction of the Δ*waaJ*::Cm mutation into mOMV producing strain, LPS from waaJ-mOMV was uniformly truncated (lane 2) due to lack of attachment of the O157-antigen polymer compared with the mOMV producing strain (lane 1). (B) Lack of WaaJ protein (an α-1,2-glucosyltransferase involved in the LPS core biosynthesis [13]) confers the EHEC O157:H7 strain with truncated LPS consisting of lipid A with a core oligosaccharide extended to the galactose residue. The lipid A portion of LPS lacks the secondary myristate chain (red-colored) of the mOMV producing strain due to a MsbB deficiency (lipid A acyltransferase [15]).

Characteristic molecular patterns of LPS were visualized by SDS-PAGE separation and silver-staining of samples extracted from mOMV and waaJ-mOMV producing (EHEC O157:H7-waaJ) strains. Because of the Δ*waaJ*::Cm mutation, LPS of the waaJ-mOMV producing strain was uniformly truncated (lane 2, Figure 1A) due to lack of attachment of the O157-antigen polymer compared to LPS of the mOMV producing strain (lane 1, Figure 1A). Likewise, the lipid A portion of LPS lacked a secondary myristate chain (red-colored) in the mOMV producing strain due to deficiency of MsbB activity (lipid A acyltransferase [15], Figure 1B). Similarly, loss of WaaJ activity (an α-1,2-glucosyltransferase involved in the LPS core biosynthesis [13]) rendered the waaJ-mOMV producing strain with truncated LPS consisting of lipid A with the core oligosaccharide extended to the galactose residue (Figure 1B).

### Characterization of EHEC O157:H7-Derived mOMVs and waaJ-mOMVs

**Figure 2:**
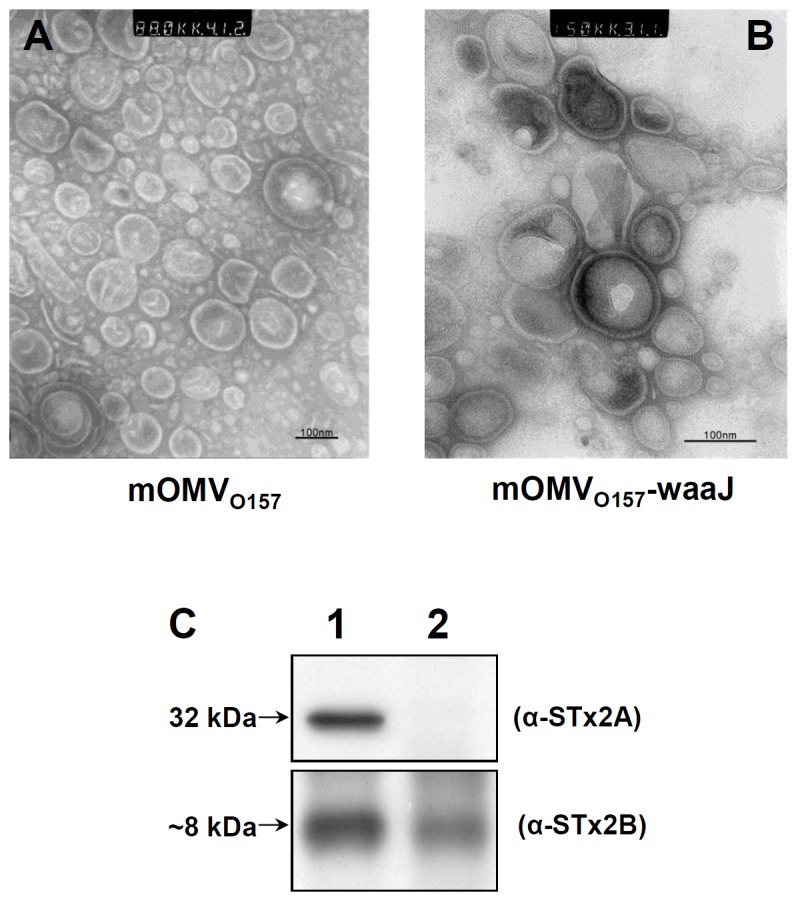
Characterization of modified OMVs isolated from EHEC O157:H7 mutants. (A, B) EHEC O157:H7-derived mOMVs were characterized by TEM and immunoblot (IB) analysis. The two mOMVs (mOMV and waaJ-mOMV) visualized by TEM exhibited the expected shape and size, suggesting that waaJ-mOMVs with uniformly truncated LPS were more heterogeneous than mOMVs. (C) IB analysis of OMVs with monoclonal antibodies (anti-STx2A and anti-STx2B) revealed that the mOMVs (lane 2) were STxA-deficient, but retained STxB subunits compared to the OMVs of parental Sakai-DM strain (lane 1), which produces less endotoxic form of LPS [5].

Next, EHEC O157:H7-derived mOMVs were characterized by TEM and immuno-blot (IB) analyses. Comparison of the shape and size of the two mOMV types suggested that the waaJ-mOMV (Figure 2B) containing a uniformly truncated LPS was more heterogeneous in size formation than that of mOMV (Figure 2A). IB analysis of both mOMV types with monoclonal antibodies (anti-STx2A and anti-STx2B) showed that mOMVs were STxA-deficient but retained STxB subunits (lane 2, Figure 2C). To confirm the immunogenicity of the STxA-deficient mOMVs, we vaccinated mice with the mOMVs intramuscularly and it showed significant increase of total IgG anti-mOMV antibody production in mice (Figure S1).

### Systemic and Mucosal Antibody Responses

**Figure 3:**
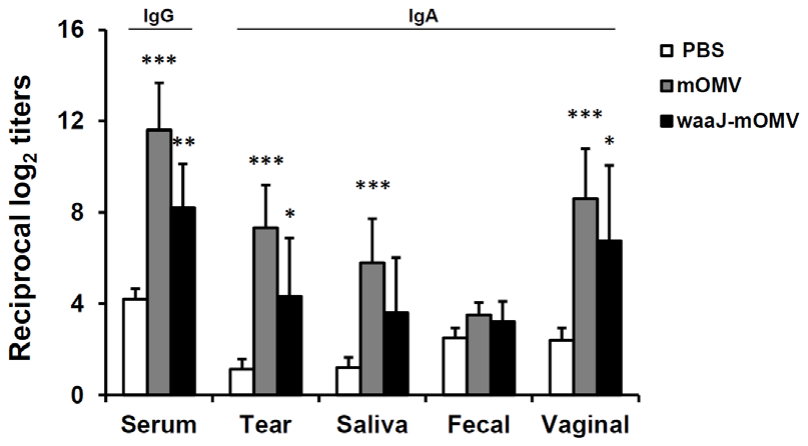
Eyedrop vaccination of mOMVs and waaJ-mOMVs resulted in both systemic and mucosal immune responses. Groups of BALB/c mice received 10 µg of the mOMVs or waaJ-mOMVs resolved in 5 µl PBS or PBS by eyedrop on both eyes twice at a 2-week interval. mOMVs and waaJ-mOMVs-specific antibody titers were measured by ELISA in serum and in various mucosal secretions at 2 weeks after final vaccination. Results are representative of three independent experiments, with five mice in each experimental group. *p<0.05, **p<0.01, ***p<0.001 compared with the PBS group.

To assess the immunogenicity of eyedrop mOMVs and waaJ-mOMVs vaccination, groups of mice were administered with mOMVs and waaJ-mOMVs by eyedrop instillation. As shown in Figure 3, serum IgG Ab and mucosal IgA Ab levels in all vaccinated mice were significantly increased than PBS group. Although the levels of both total Ag-specific IgG and IgA Ab of mOMVs vaccinated mice were not significantly higher than that of waaJ-mOMVs vaccinated mice, mOMVs immunization induced slightly higher Ab production levels than waaJ-mOMVs vaccination (Figure 3). These results indicate that the LPS O-antigen in mOMVs may serve as a strong immunogenic component capable of eliciting an antibody response towards eyedrop mOMVs vaccine. Furthermore, induction of significant enhancement of systemic IgG and mucosal IgA antibody production by vaccination of eyedrop mOMVs alone without any addition of adjuvant suggested that mOMV nano-particles could be effective vaccination vehicles by providing major antigens (LPS and outer membrane proteins) in their native form without additive adjuvants. Additionally, the formation of serum IgG antibodies cross-reactive to wtOMVs was confirmed in both mOMVs and waaJ-mOMVs vaccinated groups, and there was significantly higher level of mOMVs-specific antibody that cross-reacted to wtOMVs, but not in the waaJ-mOMVs group (Figure S1).

**Figure 4:**
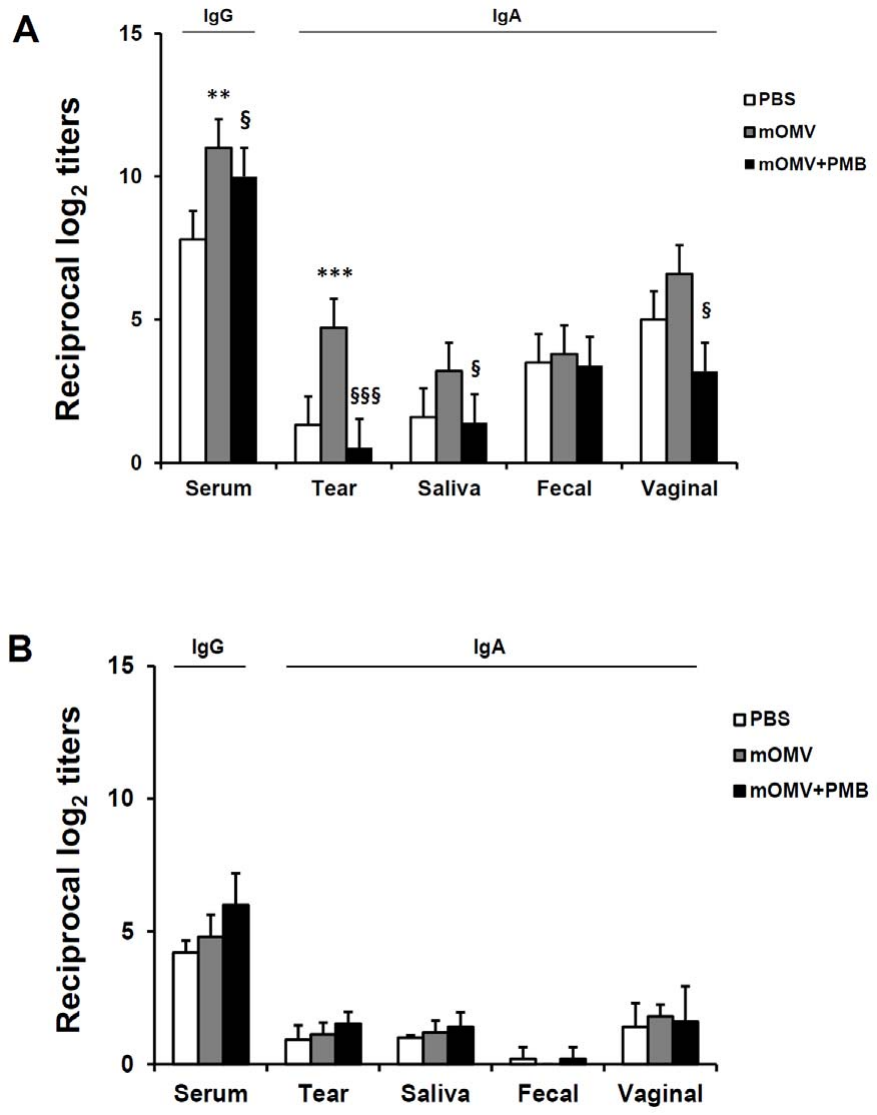
Eyedrop vaccination of mOMVs plus PMB blocked immune responses in mice. Groups of mice given the 10 µg of mOMVs alone or plus 10 µg of PMB resolved in 5 µl of PBS by eyedrop on each eye twice at a 2-week interval. mOMVs- (A) or Anti-LPS_O157_-specific antibody titers (B) were measured by ELISA in serum or in various mucosal secretions at 2 weeks after final vaccination. Results are representative of three independent experiments, with five mice in each experimental group. **p<0.01, ***p<0.001 compared with the PBS group; §*p*<0.05, §§§*p*<0.001 compared with mOMV-vaccinated group.

In this respect, we next evaluated whether premixing mOMVs with polymyxin B (PMB), which is known as an endotoxin neutralizing drug, would decrease the immunogenic potential of mOMVs. As shown in Figure 4, PMB treatment significantly down-regulated the levels of serum IgG and mucosal IgA Ab in mice vaccinated with eyedrop mOMVs (Figure 4A). However, the levels of LPS-specific antibodies were not changed after the addition of PMB (Figure 4B). These results suggest that the LPS component of mOMVs administered via the eye mucosa can serve not only as a vaccine antigen itself, but also a natural form of strong adjuvant within mOMV vesicle.

### Protection Efficacy Assessed for Eyedrop Vaccination of the mOMVs

**Figure 5:**
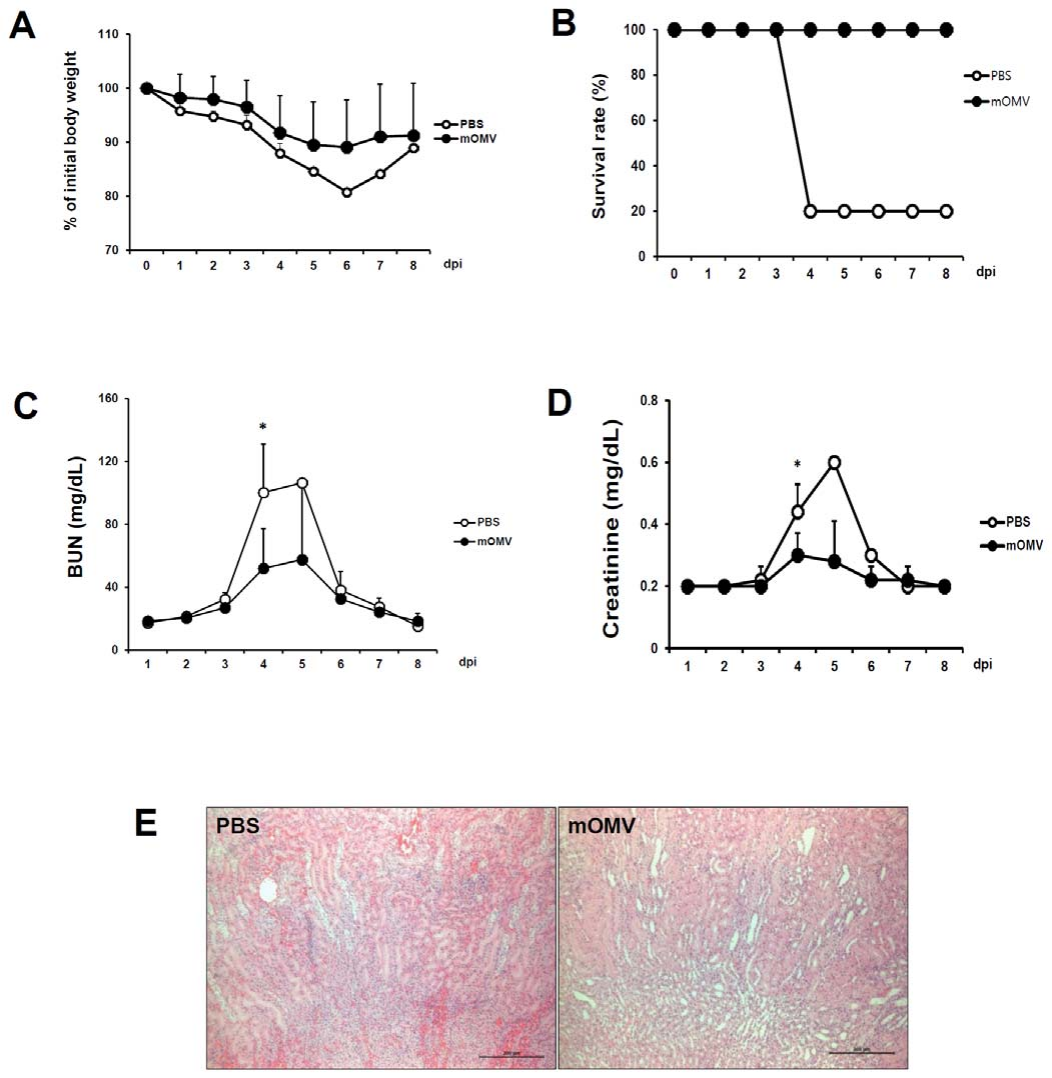
Eyedrop vaccination of mOMVs can protect vaccinated mice from wtOMVs challenge. For the protection assay, groups of BALB/c mice vaccinated with 10 µg of the mOMVs resolved in 5 µl PBS or PBS by eyedrop on both eyes twice at a 2-week interval. At 2 weeks after final vaccination, mice were injected with 1.5X LD_50_ of wild-type EHEC O157:H7 OMVs (LD_50_ 0.274 mg/kg) intraperitoneally. Body weights (A) and survival rates (B) were monitored daily. For the measurement of BUN (C) and creatinine (D) levels as indicators of mouse renal function, serum samples were acquired from all challenged mice daily. (E) For the assessment of renal failure by histological observation, kidney tissues were sampled from survived mice of each group at 4 days post challenge and stained with H&E staining (original magnification×100). Results are representative of three independent experiments, with five mice in each experimental group. *p<0.05 versus PBS-vaccinated group.

To assess the vaccine efficacy of eyedrop immunization of mice with mOMVs, a challenge experiment was performed by intraperitoneal injection of lethal dose (1.5X LD_50_) of wtOMVs. Mice vaccinated with mOMVs exhibited a slight loss of body weight (Figure 5A) and were completely protected from challenge with wtOMVs compared to the PBS control group (Figure 5B). We next analyzed BUN and Cr levels in serum of challenged mice. The serum levels of BUN and Cr in the mOMVs-vaccinated group were not significantly elevated compared with the control group (Figure 5C and D), and these were consistent with histological observations of the renal tissues of mice vaccinated with mOMVs which showed virtually no sign of renal bleeding compared to that of PBS group (Figure 5E, right panel). Thus, when results above were considered together, the eyedrop mOMVs-vaccination induced systemic antibody production against the components of mOMVs and it binds and neutralizes peritoneally injected wtOMVs so that Shiga toxins within wtOMVs were blocked in advance and cleared together with wtOMVs.

### Safety of mOMVs Administered by Eyedrop

**Figure 6:**
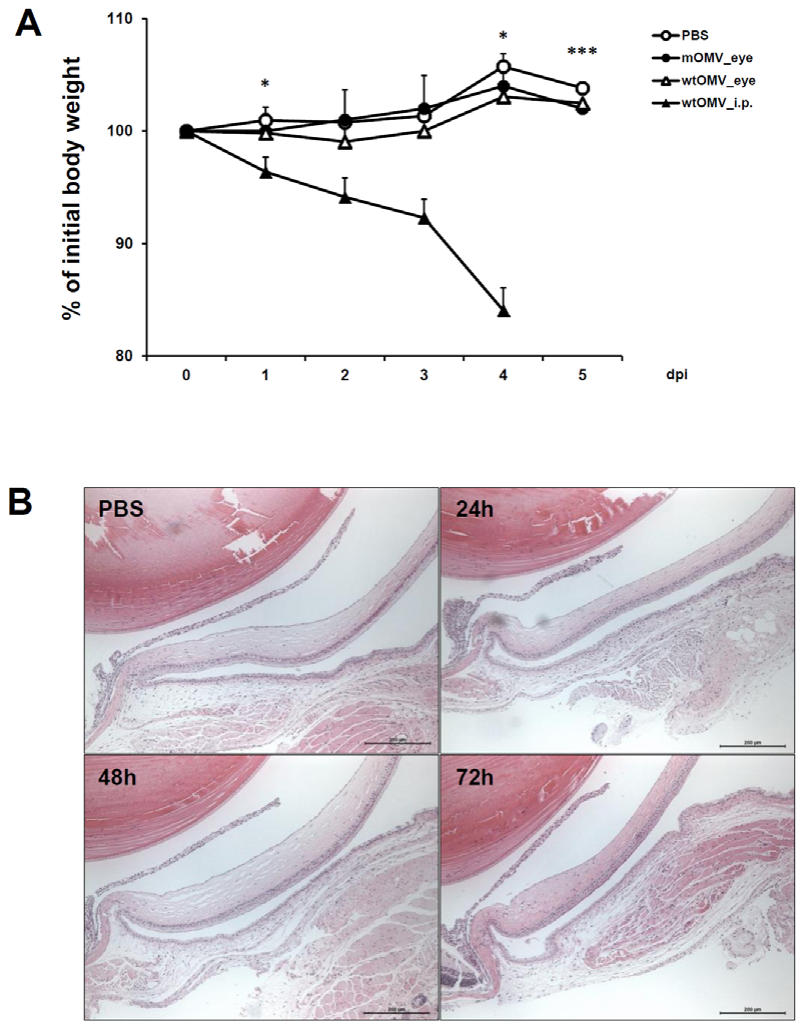
Safety evaluation of eyedrop mOMVs in mice. Groups of BALB/c mice were administered with 10 µg of mOMVs or wtOMVs resolved in 5 µl of PBS by eyedrop on each eye or 10 µg of wtOMVs resolved in 100 µl of PBS intraperitoneally. (A) Body weight changes were monitored on a daily basis for 5 days. (B) Eye tissues were acquired at 24, 48 and 72 h after administration of 10 µg of mOMVs for histologic examination (H&E, original magnification X100). As a control, PBS was administrated via eyedrop. *p<0.05, ***p<0.001 between ocular wtOMV-treated group and intraperitoneally wtOMV-treated group.

Since the LPS components that present in the mOMVs would have a potential to induce an inflammation in administered eyes of mice, we checked the possibility of toxicity of eyedrop mOMVs. Mice were administered with 10 µg mOMVs by eyedrop and were monitored for several days. As shown in Figure 6, there was no decrease in body weight (Figure 6A) and no histopathological changes in eye mucosa (Figure 6B). Surprisingly, the administration of 10 µg wtOMVs by eyedrop induced no significant reduction on mice body weight compared to PBS treated mice, whereas mice that were peritoneally injected with the same amount (10 µg) of wtOMVs exhibited a severe loss of body weight and eventually died at 4 days post-injection (Figure 6A). Taken together, these results suggest that vaccination of eyedrop mOMVs has no toxic effect on eyes, and eye mucosa is an efficient and safe vaccine delivery route for inducing protective anti-HUS immunity.

## Discussion

We previously showed that OMVs produced from EHEC O157:H7 Sakai strain carried both Shiga toxins and LPS moieties, and that these vesicles (wtOMVs) caused HUS-like symptoms in a mouse model [12]. Thus, OMVs are now recognized as a natural HUS-causative agent produced within the guts of patients after infection with EHEC O157. Considering that EHEC O157 bacteria are not invasive, it is interesting to note that no mechanism other than the proposed OMV package delivery has been demonstrated for trans-migration of Shiga toxins and the LPS from the gut lumen of HUS patients into the blood stream [12]. In this study, we showed that eyedrop vaccination of mOMVs (engineered to be a safe vaccine) was effective for preventing HUS pathogenesis in mice challenged with HUS-causative wtOMVs. Furthermore, we also showed that loss of LPS O-antigen in the waaJ-mOMVs rendered the vesicles much less immunogenic, suggesting that the presence of LPS O-antigen in mOMVs is as an important immunogen for inducing effective anti-OMV_O157_ antibodies.

For the development of an effective vaccine against EHEC-related pathogenicity [16], [17], STxB subunit-based immunogens that have ability to induce anti-Shiga toxin antibodies have been investigated by several laboratories [18]–[20]. However, no vaccine able to prevent EHEC infection and/or STx-mediated HUS for humans and animals has been reported [18], [21]. Accordingly, we looked into what was involved in the immune reaction among mOMVs including STx2B. In the preliminary results, we could not raise detectable anti-STxB antibodies by the mOMV vaccination, although anti-STx antibody plays an important role in the prevention of HUS, presumably by neutralizing the toxins directly. However, the immune responses raised by the mOMV vaccination could protect the mice from the lethal challenge with wtOMVs given intraperitoneally. This finding supports our previous report [12] that STx toxins are mostly enclosed within the OMVs that carry the cargo from the gut where STx is produced to the blood stream (kidney targeting). Therefore, it is likely that anti-mOMV antibodies raised by eyedrop vaccination can block the spread of the toxin by preventing the OMV translocation and/or circulation into the blood stream.

Mucosal vaccination has numerous merits and thus has gained a great deal of attention. Specifically, mucosal vaccination is potentially more convenient and inexpensive compared with parenteral vaccines, in that it does not require specialized skill for administration, and can stimulate secretion of IgA antibodies capable of neutralizing pathogens at sites of entry into the body [22]. Heat-labile toxin (LT) of enterotoxigenic *E.coli* (ETEC) is well known mucosal vaccine adjuvant; however, intranasal vaccination with LT-adjuvanted vaccine has raised safety concerns regarding the nervous system [23]. Mucosal vaccination using an ocular route [24]–[26] has been shown to effectively protect host against diverse array of pathogens [27]–[30]. Since OMVs of enterotoxigenic *E. coli* have been reported to induce protective immune responses against colonization of ETEC strains in the murine intestine [31], we attempted to determine whether eyedrop vaccination of mOMVs from EHEC O157 could effectively prevent HUS after pathogenic *E.coli* attack. But eyedrop vaccination of the mOMVs could not raise enough gut-mucosal immune responses required for lowering the intestinal colonization by the EHEC O157. Instead, it could induce protective immune responses preventing systemic distribution of the STx-containing OMVs. In the context of local gut-associated mucosal antibody response, it is worth attempting to test and compare the effects of mucosal vaccination performed via nasal, sublingual, and oral routes in the further study. Additionally, enhancing the mucosal immunity through the addition of optimal adjuvants, the usability of mOMVs as the effective mucosal vaccine would be more increased.

The experimental eyedrop vaccine model used in this study was intended to test whether the mOMV could induce mucosal and/or systemic immune responses or not, which is enough to protect the vaccinated animal from the lethal HUS-causative agent (wtOMVs), for the first time, via ocular route of immunization. Therefore, the intestinal colonization model was not employed in this study to evaluate the mOMV-mediated mucosal vaccine effectiveness against the EHEC O157 infection in the gut. It will also be interesting to test whether mOMVs derived from the Sakai-O157 strain can act as an effective vaccine against heterologous EHEC infections. However, our experiment has given the prioriy in checking out both the formation of neutralizing antibody against mOMVs and waaJ-OMVs and the protection against wtOMVs. Although, the exposure of the R3-core in the LPS lacking the O157 antigen in the waaJ-mOMV can elicit the R3-core-specific anti-LPS antibodies that may cross-react to non-O157 Shiga toxin-producing *E. coli* (STEC) pathogens possessed with R3-type LPS core commonly [32], we did not test this possibility that would be another theme to be pursued in our further experiment.

In addition to mOMVs, which consist of the much-less endotoxic LPS (penta-acylated lipid A) and are devoid of STxA subunits, we tested the immunogenicity of waaJ-mOMVs, which are devoid of the O-antigen side chains of O157 LPS in the mOMV backbone. O-antigen truncation of LPS of bacterial pathogens is often associated with decreased immunogenicity compared with full-length LPS [33]. But, sometimes, exposure of the core oligosaccharides with O-antigen truncated LPS is supposed to provide additional immunogenicity [34]. In the case of waaJ-mOMVs (Figure 3), O-antigen truncation rendered the vesicles less immunogenic than the ocular mOMV vaccination model, suggesting that O-antigen in mOMVs may serve as a strong immunogenic component capable of eliciting an antibody response towards the mOMV vaccine. Furthermore, PMB treatment of mOMVs, which was used to block the LPS-mediated adjuvant activity of mOMVs [35], resulted in a significant reduction of anti-OMV_O157_ antibody titers (Figure 4A). Interestingly, the ability for inducing anti-LPS_O157_ antibody was not affected by PMB treatment (Figure 4B). Since PMB binding to LPS of mOMVs by charge-to-charge interactions can block TLR-4 dependent innate immunity activation pathways [36], it has been suggested that PMB treatment may decrease the potential adjuvant activity of LPS within mOMVs administered to the ocular mucosa. Based on our results, LPS moiety of mOMVs served as an adjuvant by promoting immunogenicity of vesicle antigens, but did not contribute towards increased production of anti-LPS antibodies.

In conclusion, eyedrop vaccination using mOMVs of EHEC O157 bacteria demonstrated for the first time that mOMVs are efficient vaccination tools which do not require additional adjuvants. Moreover, eyedrop vaccination with mOMVs was shown to be effective for preventing HUS pathogenesis in mice against challenge with HUS-causative wtOMVs. We also demonstrated that loss of LPS O-antigen of waaJ-mOMVs rendered vesicles much less immunogenic compared to mOMVs. Thus, taken together, these results suggest that mOMVs can be utilized as a safe ocular-mucosal vaccine capable of inducing a protective immune response against HUS-causative OMVs of EHEC O157 bacteria.

## Supporting Information

Figure S1 **Eyedrop vaccination of mOMVs** **resulted in wtOMVs cross-reactive Ab production.** Groups of BALB/c mice received 10 µg of the mOMVs or waaJ-mOMVs resolved in 10 µl PBS or PBS alone by eyedrop on both eyes twice at a 2-week interval. wtOMVs cross-reactive antibody titers were measured by ELISA in serum at 2 weeks after final vaccination. Results are representative of three independent experiments, with five mice in each experimental group. *p<0.05 compared with the PBS group.
(ZIP)
