# Supplementary figures and images for: Protection from Hemolytic Uremic Syndrome by Eyedrop Vaccination with Modified Enterohemorrhagic E. coli Outer Membrane Vesicles
Source: PLoS One. 2014 Jul 17;9(7):e100229. doi: 10.1371/journal.pone.0100229 (PMC4102476; doi:10.1371/journal.pone.0100229)

## Slide 1
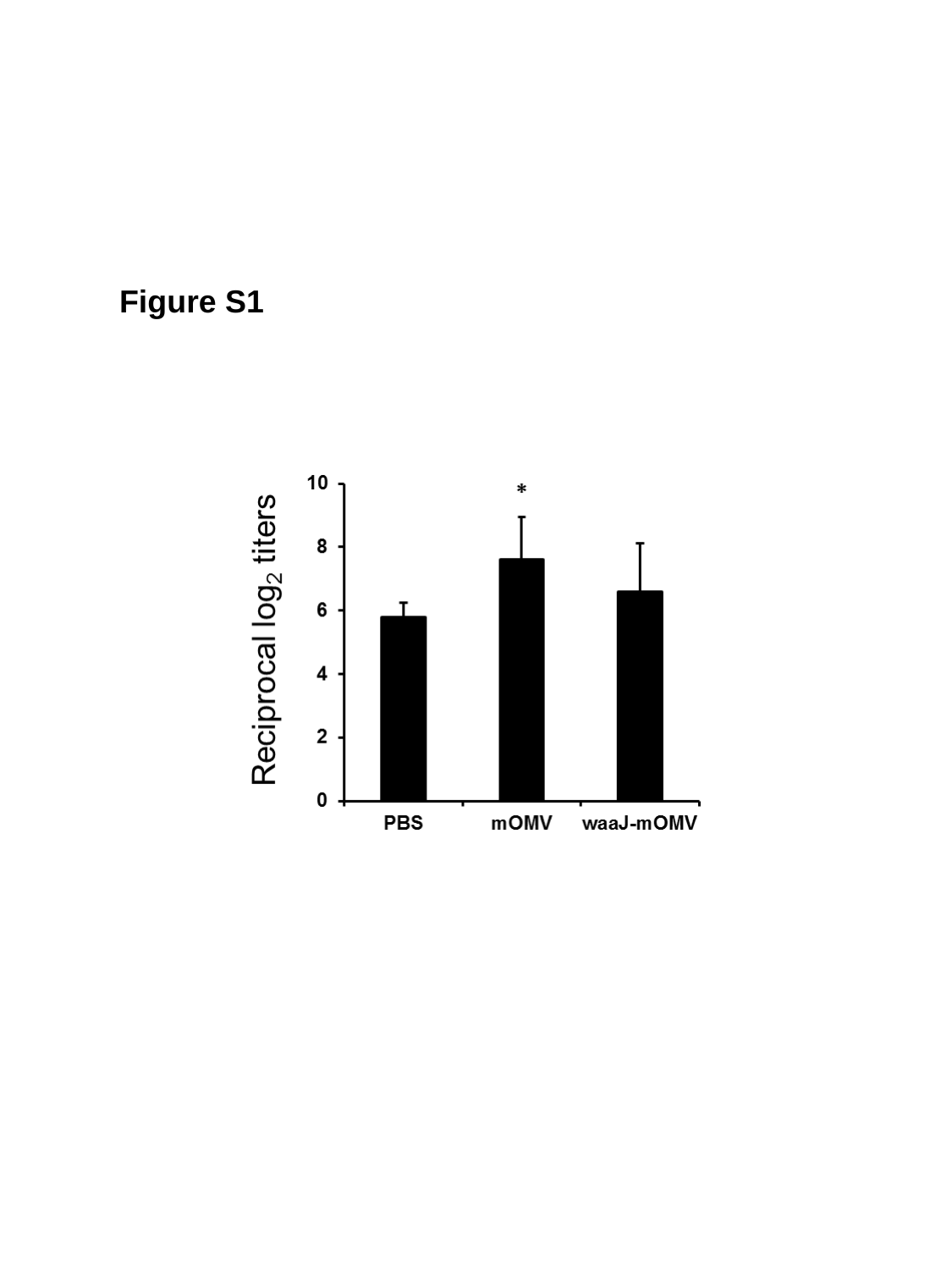

Figure S1

Supplement: Figure S1 — Eyedrop vaccination of mOMVs resulted in wtOMVs cross-reactive Ab production. Groups of BALB/c mice received 10 µg of the mOMVs or waaJ-mOMVs resolved in 10 µl PBS or PBS alone by eyedrop on both eyes twice at a 2-week interval. wtOMVs cross-reactive antibody titers were measured by ELISA in serum at 2 weeks after final vaccination. Results are representative of three independent experiments, with five mice in each experimental group. *p<0.05 compared with the PBS group. (ZIP) [file pone.0100229.s001.zip › Figure S1.pptx]

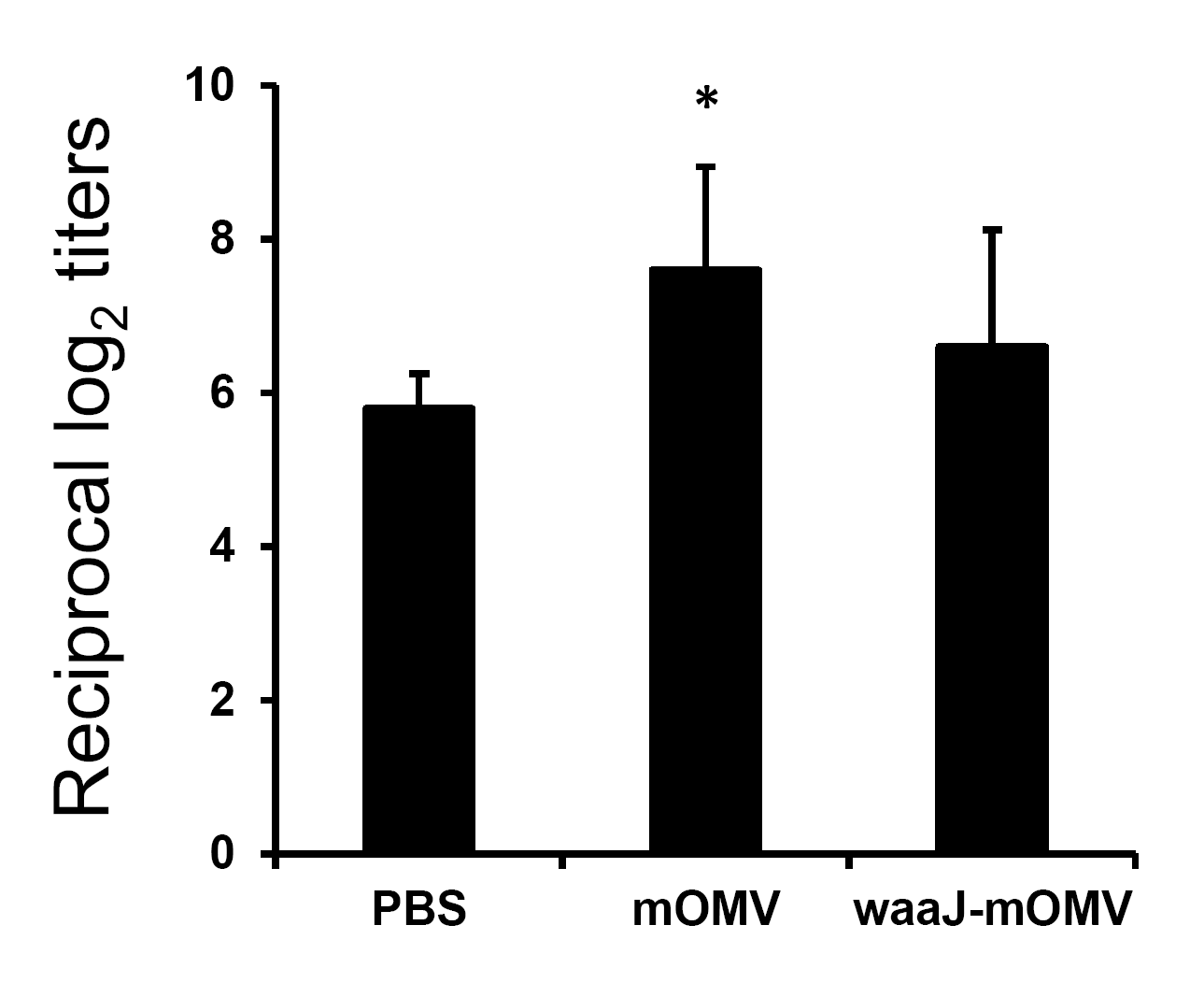

Supplement: Figure S1 — Eyedrop vaccination of mOMVs resulted in wtOMVs cross-reactive Ab production. Groups of BALB/c mice received 10 µg of the mOMVs or waaJ-mOMVs resolved in 10 µl PBS or PBS alone by eyedrop on both eyes twice at a 2-week interval. wtOMVs cross-reactive antibody titers were measured by ELISA in serum at 2 weeks after final vaccination. Results are representative of three independent experiments, with five mice in each experimental group. *p<0.05 compared with the PBS group. (ZIP) [file pone.0100229.s001.zip › Figure S1.tif]
